# Microbiomic and Metabolomic Insights into the Mechanisms of Alfalfa Polysaccharides and Seaweed Polysaccharides in Alleviating Diarrhea in Pre-Weaning Holstein Calves

**DOI:** 10.3390/ani15040485

**Published:** 2025-02-08

**Authors:** Jianan Zhao, Haoliang Tian, Xiaohui Kong, Danqi Dang, Kaizhen Liu, Chuanyou Su, Hongxia Lian, Tengyun Gao, Tong Fu, Liyang Zhang, Wenqing Li, Wei Zhang

**Affiliations:** Henan International Joint Laboratory of Nutrition Regulation and Ecological Raising of Domestic Animal, College of Animal Science and Technology, Henan Agricultural University, Zhengzhou 450046, China; zhaojianan166@163.com (J.Z.); 17772133035@163.com (H.T.); kongxiaohui2024@163.com (X.K.); dangdanqi@163.com (D.D.); liukaizhen@henau.edu.cn (K.L.); suchuanyou@henau.edu.cn (C.S.); lhx263@sina.com (H.L.); dairycow@163.com (T.G.); futong2004@126.com (T.F.); zhangliyang@henau.edu.cn (L.Z.)

**Keywords:** alfalfa polysaccharides, calves’ diarrhea, growth performance, gut microbes, seaweed polysaccharides, nontargeted metabolomics

## Abstract

Neonatal calves’ diarrhea, which can be severe enough to cause death, has a significant impact on the global cattle industry. In this study, alfalfa polysaccharides and seaweed polysaccharides were found to significantly improve the diarrhea condition in neonatal calves. To explore the underlying mechanisms, further microbiomic and metabolomic analyses were conducted. This study investigated the impact of alfalfa polysaccharides and seaweed polysaccharides on growth performance, serum metabolites, gut microbiota, and metabolomics in neonatal Holstein calves. A total of 24 newborn calves were randomly assigned to three groups, with 8 calves per treatment group. The control (CON) group was fed a basal diet, the alfalfa polysaccharide (AP) group received a basal diet supplemented with alfalfa polysaccharides (4 g/calf/day), and the seaweed polysaccharide group (SP) received a basal diet supplemented with seaweed polysaccharides (4 g/calf/day). These polysaccharides were plant extracts. Compared to the CON group, the results indicated that SP significantly enhanced the body weight, height, chest circumference, and average daily gain of Holstein calves (*p* < 0.05), while also reducing the diarrhea rate and improving manure scoring (*p* < 0.05). Compared to the CON, AP also reduced the diarrhea rate (*p* < 0.05). In terms of serum biochemistry, supplementation with AP and SP increased serum alkaline phosphatase (ALP) and insulin-like growth factor 1 (IGF-1) levels compared to the CON group (*p* < 0.05). Both AP and SP elevated serum catalase (CAT) and Total Antioxidant Capacity (T-AOC) levels, indicating enhanced antioxidant status (*p* < 0.05). Regarding immune responses, supplementation with AP and SP significantly increased serum complement component 3 (C3) and immunoglobulin M (IgM) levels, while significantly reducing pro-inflammatory cytokines interleukin-18 (IL-18), tumor necrosis factor alpha (TNF-α), and interferon-gamma (IFN-γ) compared to the CON group (*p* < 0.05). Microbiota analysis revealed that AP modulated the abundance of *Firmicutes*, while SP influenced the abundance of *Prevotella* and *Succiniclasticum*. AP and SP differentially influenced intestinal metabolites compared to the CON group, leading to enrichment in pathways related to immunity, antibacterial, and anti-inflammatory functions. These pathways included the biosynthesis of alkaloids from ornithine, lysine, and nicotinic acid, glucocorticoid and mineralocorticoid receptor canothersis/antagonists, secondary metabolite biosynthesis, and alkaloid biosynthesis from histidine and purine, thus alleviating intestinal inflammation. Therefore, by supplementing with AP and SP, the diarrhea rate in calves was reduced, and the immune function of Holstein calves was enhanced, while simultaneously promoting a higher relative abundance of beneficial gut bacteria and suppressing the relative abundance of pathogenic bacteria. Additionally, gut pathways associated with immune response and inflammation were modulated by AP and SP. This study provided valuable insights and theoretical underpinnings for the use of AP and SP in preventing diarrhea in neonatal calves.

## Simple Summary

In this study, it was discovered that feeding alfalfa polysaccharides and seaweed polysaccharides reduced the incidence of diarrhea and enhanced immune function in Holstein calves. To investigate this phenomenon, microbiomic and metabolomic analyses were further employed to elucidate the underlying mechanisms. The levels of serum catalase and Total Antioxidant Capacity were increased by these polysaccharides, indicating an enhanced antioxidant state. In terms of immune response, the levels of serum complement component 3 and immunoglobulin M were elevated, while pro-inflammatory cytokines were reduced. Gut pathways associated with immunity, antimicrobial, and anti-inflammatory functions were modulated by these polysaccharides, significantly alleviating intestinal inflammation. Additionally, the relative abundance of beneficial bacteria was increased, while pathogenic bacteria were suppressed.

## 1. Introduction

Neonatal calves’ diarrhea is prevalent on farms worldwide, adversely affecting animal health, welfare, and farm profitability [1]. Factors such as intestinal inflammation, tumor infiltration, and gut microbiota dysbiosis contribute to neonatal calf diarrhea [2]. These factors collectively disrupt gut function, leading to impaired nutrient absorption and the invasion of harmful microorganisms and toxins, such as *Escherichia coli* [3], heat-stable toxins, and endotoxins [4], resulting in diarrhea. Intestinal inflammation could damage the gut mucosa, blood vessels, and lymphatic system, increasing gut permeability, which causes protein and blood leakage, exacerbating malnutrition and reducing immunity [5]. Dysbiosis of the gut microbiota leads to a reduction in beneficial bacteria and an increase in pathogenic bacteria, disrupting the balance of the gut microbiota and affecting the normal absorptive and secretory functions of the gut [6]. For example, beneficial bacteria, such as *Lactobacillus*, *Bifidobacterium*, and *Streptococcus thermophilus*, decrease in levels [7]. On the other hand, pathogenic bacteria, including Escherichia coli and Salmonella spp., increase in levels, causing severe gastrointestinal issues [8].

Maternal sources are the primary origin of beneficial gut microorganisms in newborn calves, as calves acquire these bacteria during birth and through contact with their mother’s skin, milk, and feces [9]. Environmental exposure and dietary intake (feed and milk) also contribute beneficial bacteria [9]. Prenatal colonization through translocation from maternal sources may start in the womb and continue via exposure to beneficial microbes during birth and ingesting colostrum and milk postpartum [10]. The initial microbiota is both innate and acquired, rapidly diversifying as calves interact with their surroundings and diet. Seaweed polysaccharides are utilized by *Bifidobacterium* spp. as prebiotics [11], while alfalfa polysaccharides are utilized by *Lactobacillus* spp. [12].

The use of antibiotics to prevent diarrhea in newborn calves has led to issues such as antibiotic resistance, as well as disruption in the establishment of early-life microbiota [13]. Therefore, natural bioactive substances have been investigated by scientists as alternatives. Natural polysaccharides such as alfalfa polysaccharides (APs) and seaweed polysaccharides (SPs) have garnered widespread attention due to their rich bioactive components and potential health benefits [14,15]. AP, which is extracted from alfalfa, has been found to exhibit various bioactivities including anti-inflammatory, antioxidant, and immunomodulatory effects [14]. Research has shown that AP activates splenic B cells through TLR4, primarily exerting its immune functions via the MAPK and NF-κB signaling pathways [16]. SP, which is extracted from various seaweeds, possesses multiple functions including antibacterial, antiviral, antioxidant, antitumor, and immunomodulatory effects [17]. Studies have indicated that seaweeds and their derived metabolites hold potential in aquaculture feed, particularly in enhancing fish immune responses and gut health [18].

The mechanisms of complex diseases have been revealed through multiomics analysis, which also enhanced the understanding of the interactions among various substances in the body and their biological significance [19]. Few studies have used multiomics techniques to investigate the effects of AP and SP on diarrhea in neonatal Holstein calves. This study analyzed the growth performance, serum metabolites, gut microbiota, and gut metabolites of neonatal Holstein calves fed with AP and SP, exploring the processes and mechanisms by which AP and SP regulate diarrhea in neonatal Holstein calves. The findings aim to provide data references for the application of these polysaccharides in calf health management.

## 2. Materials and Methods

### 2.1. Experimental Materials

The extraction process for alfalfa polysaccharides was as follows [14]: Fresh alfalfa samples were cut, dried at 65 °C, and mixed with distilled water in a ratio of 1:10. After boiling for 1 h, the mixture was filtered and centrifuged at 1000× *g* for 10 min. The supernatant was mixed with 4 volumes of anhydrous ethanol and left at 4 °C for 12 h. It was then centrifuged at 3000× *g* for 10 min to precipitate crude polysaccharides. These were dissolved in distilled water, dialyzed at 4 °C for 2 days, and deproteinized twice with chloroform–butanol. Finally, the precipitate was collected and freeze-dried to obtain purified polysaccharides. The alfalfa polysaccharides was provided by Fufeng Snot Bio-Technology Co., Ltd. (Baoji, China).

The extraction process for seaweed polysaccharides was as follows [15]: Seaweed powder was soaked in distilled water for 2 h at room temperature, homogenized, and refluxed at 100 °C for 2 h. The mixture was filtered and centrifuged, and the supernatant was extracted three more times with distilled water at 100 °C for 2 h each time. All extracts were combined, concentrated, and dialyzed for three days using a cellulose membrane and then freeze-dried. The polysaccharides were dissolved, treated multiple times with Sevag reagent, precipitated with ethanol at 4 °C overnight, centrifuged, washed, and freeze-dried to obtain purified polysaccharides. The seaweed polysaccharides was provided by Rongcheng Hongde Marine Bio-Technology Co., Ltd. (Nipomo, CA, USA).

### 2.2. Animal Experiment

The animal experimental procedures were approved by the Animal Ethics Committee of Henan Agricultural University (No: HNND2024031237). Twenty-four healthy Holstein calves, with an average weight of 338.10 ± 3.74 kg (mean ± standard deviation) and an average age of 4.12 ± 2.33 days (mean ± standard deviation) were selected for this study. At the beginning of the experiment, each calf was fed three times a day with 2 L of milk replacer per feeding. The calves were randomly assigned to three groups: the control (CON) group, which was fed a basal diet; the alfalfa polysaccharide (AP) group, which was fed a basal diet plus alfalfa polysaccharides (4 g/calf/day); and the seaweed polysaccharide group (SP), which was fed a basal diet plus seaweed polysaccharides (4 g/calf/day). The composition of the calf feed and the nutritional levels of the milk replacer were analyzed according to AOAC methods [20]; detailed information is provided in Table 1. Initially, all calves were fed 5 L of milk replacer daily, which was later increased to 6 L per day. The ingredients of the milk replacer powder included whole milk powder, skim milk powder, concentrated whey protein, whey powder, demineralized whey powder, lactose, coconut oil, palm oil, trace elements, vitamin complex, lysine, and methionine.

### 2.3. Sample Collection

Fecal scores and diarrhea rates were recorded for the calves. Fecal scoring was based on stool consistency and the presence of blood. Normal stool with no bleeding was scored 1. Soft and shaped stool with slight bleeding was scored 2. Meager stool with moderate bleeding was scored 3. Watery stool with visible bleeding was scored 4. When a fecal score of ≥ 3 was recorded, the stool was classified as diarrhea. The diarrhea rate was calculated using the following formula [21]. On days 0 and 56 of the trial, the calves were weighed, and measurements of height, body length, diagonal body length, and chest circumference were taken, along with feed intake. On day 56, blood samples were collected from the calves for subsequent analysis of serum biochemistry, antioxidant, and immune parameters. Additionally, calf feces were collected for microbiome and metabolome analyses.

$$Diarrhea\;Rate\;(\%)=\frac{Total\;Number\;of\;Diarrhea\;Incidents\times Number\;of\;Diarrhea\;Days}{Total\;Number\;of\;Calves\times Number\;of\;Experimental\;Days}\times 100$$

### 2.4. Detection of Serum Metabolites

The measurements of IL-1β (interleukin-1 beta), IL-4 (interleukin-4), IL-18 (interleukin-18), TNF-α (tumor necrosis factor-alpha), IFN-γ (interferon-gamma), IgA (immunoglobulin A), IgG (immunoglobulin G), IgM (immunoglobulin M), C3 (complement component 3), C4 (complement component 4), GH (growth hormone), and IGF-1 (insulin-like growth factor 1) were conducted with the assistance of Jiangsu Meimian Biotechnology Co., Ltd. (Nantong City, China). Kits for these measurements were provided by Jiangsu Meimian Industry Co., Ltd. (Changzhou, China). The immunoglobulins were measured using a Swiss Sunrise (Opfikon, Switzerland) automatic ELISA reader (F50, CH) via immunoturbidimetry.

The measurements of SOD (superoxide dismutase), MDA (malondialdehyde), T-AOC (total antioxidant capacity), and CAT (catalase) were conducted with the assistance of Beijing Huaying Biotechnology Institute. Kits for these measurements were obtained from Jiangsu Edson Biotechnology Co., Ltd. (Nanjing, China).

The measurements of TP (total protein), ALT (alanine aminotransferase), AST (aspartate aminotransferase), ALB (albumin), ALP (alkaline phosphatase), and LDH (lactate dehydrogenase) were conducted with kits from Shandong Boke Biological Industry Co., Ltd. (Jinan, China). These measurements were performed using an automatic biochemical analyzer (BK-280, Shandong Boke Biological Industry Co., Ltd., Jinan, China).

The experimental procedures for all the aforementioned indicators were conducted according to the instructions provided in the reagent kit’s manual.

### 2.5. 16S rDNA Gene Sequencing for Gut Microbiota Analysis

Genomic DNA of the microbial community was extracted from the intestinal contents of calves, and the V3 + V4 region of the 16S rDNA was specifically amplified. The primer sequences used were 341F (5’-CCTACGGGNGGCWGCAG-3’) and 806R (5’-GACTACHVGGGTATCTAATCC-3’). The purified amplification products were mixed and linked to sequencing adapters to construct an amplification library, which was sequenced using the Illumina PE250 platform (Illumina, Inc., San Diego, CA, USA). Based on the UPARSE algorithm, low-quality clean tag sequences were clustered. By default, sequences were clustered into ASVs at 97% similarity to obtain their abundance and representative sequences. After sequencing and obtaining raw reads, Usearch software(Version 11.0.667) was chosen. Low-quality reads were first filtered, and then paired-end reads were merged into tags. These tags were subsequently filtered for low quality, resulting in clean tags. Based on the clean tags, Usearch software(Version 11.0.667) was used to perform clustering, during which any chimeric tags detected were removed, yielding the operational taxonomic units (OTUs) and their representative sequences. Utilizing the sequence and abundance data of OTUs or amplicon sequence variants (ASVs), analyses such as species annotation, species composition, indicator species analysis, α-diversity analysis, β-diversity analysis, and community function prediction were conducted. The LEfSe (LDA effect size) analysis was used to perform comparisons between two or more groups. Dimensionality reduction and evaluation of the impact of significantly different species (the LDA score) were conducted using linear discriminant analysis (LDA), a supervised classification method. LEfSe analysis (threshold LDA > 2, *p* < 0.05) was performed using a professional platform (https://www.omicshare.com/ (accessed on 28 October 2024)).

PICRUSt2 (Phylogenetic Investigation of Communities by Reconstruction of Unobserved States 2) was utilized as a microbial functional prediction tool based on 16S rRNA gene sequence data. The core concept of PICRUSt2 involved using known reference genomes to infer the functional gene sets within a sample by constructing an evolutionary tree similar to the target sample. Compared to PICRUSt1, PICRUSt2 employs more advanced algorithms and an expanded database, significantly enhancing the accuracy of functional predictions. Functional prediction of the microbiome was conducted using PICRUSt2.

### 2.6. Untargeted Metabolomics Analysis of Fecal Samples

Analysis was performed using an UHPLC (1290 Infinity LC, Agilent Technologies, Santa Clara, CA, USA) coupled to a quadrupole time-of-flight mass spectrometer (AB Sciex TripleTOF 6600, Shanghai, China). For HILIC separation, samples were analyzed using a 2.1 mm × 100 mm ACQUIY UPLC BEH Amide 1.7 µm column (waters, Ireland). In both ESI positive and negative modes, the mobile phase contained A = 25 mM ammonium acetate and 25 mM ammonium hydroxide in water and B = acetonitrile. The gradient was 95% B for 0.5 min and was linearly reduced to 65% in 6.5 min; it was then reduced to 40% in 1 min and kept for 1 min, before being increased to 95% in 0.1 min, with a 3 min re-equilibration period employed. The ESI source conditions were set as follows: Ion Source Gas1 (Gas1) at 60, Ion Source Gas2 (Gas2) at 60, curtain gas (CUR) at 30, source temperature at 600 °C, and IonSpray Voltage Floating (ISVF) at ± 5500 V. In MS-only acquisition, the instrument was set to acquire over the *m*/*z* range 60–1000 Da, and the accumulation time for TOF MS scan was set at 0.20 s/spectra. In auto MS/MS acquisition, the instrument was set to acquire over the *m*/*z* range 25–1000 Da, and the accumulation time for product ion scanning was set at 0.05 s/spectra. The product ion scan is acquired using information dependent acquisition (IDA) with high sensitivity mode selected. The parameters were set as follows: the collision energy (CE) fixed at 35 V with ± 15 eV; declustering potential (DP) set to 60 V (+) and −60 V (−); isotopes excluded within 4 Da; and 10 candidate ions monitored per cycle. Statistical analyses included Orthogonal Projections to Latent Structures Discriminant Analysis (OPLS-DA), differential abundance metabolite identification (heatmaps, Venn diagrams, and volcano plots), and KEGG enrichment analysis of differential abundance metabolites to visualize the data.

### 2.7. Correlation Analysis Methods

The Pearson correlation coefficients between genus-level microbiota and serum metabolites were calculated using the Psych package in R (version 4.2.3). Corresponding heatmaps and network analysis graphs were generated using the Pheatmap and igraph packages in R, respectively. Additionally, the WGCNA package was employed for weighted gene coexpression network analysis of metabolites, linking serum metabolites to modules, which were subsequently enriched to KEGG pathways. Correlation analysis was conducted using a professional platform (https://www.omicshare.com/ (accessed on 28 October 2024)). The network graph, comprising nodes and edges, was constructed where nodes represented species and metabolites, while edges denoted correlations. The “node” table within the folder contained information on microbial/metabolite abundances and annotations, whereas the “edge” table stored relationship pairs, correlation coefficients, and significance levels. Both tables could be imported to cloud platforms or Cytoscape 3.10.3 software for network graph visualization and customization. Spearman correlation analysis was performed for selected differential metabolites, microbiota, and serum metabolites, and network analysis graphs were constructed using Cytoscape 3.10.3 software. A *p*-value ≤ 0.05 was considered statistically significant. The correlation coefficient (r) ranged from −1 to 1, with r > 0 indicating a positive correlation and r < 0 indicating a negative correlation.

### 2.8. Statistical Analysis

Data analysis was conducted using IBM SPSS Statistics 26 software (SPSS Inc., Chicago, IL, USA). One-way analysis of variance (ANOVA) and least significant difference (LSD) tests were employed for multiple comparisons between groups. The results were expressed as mean ± standard error (SE) and visualized using GraphPad Prism 10.0 (GraphPad Inc., La Jolla, CA, USA). Tukey’s test with a *p* < 0.05 level of significance was used for post hoc analysis to determine group differences. The significance thresholds for results were defined by confidence levels, where a 95% confidence level indicated that the results were significant if the *p*-value was less than 0.05; a 99% confidence level required *p*-values to be less than 0.01 for significance; and a 99.9% confidence level stipulated that *p*-values had to be less than 0.001 to be considered significant. Statistical significance was indicated by the following symbols: * for a significant difference (*p* < 0.05), ** for a highly significant difference (*p* < 0.01), and *** for an extremely significant difference (*p* < 0.001). The model equation is as follows:

$$Y_{ij}=\mu +\tau_{i}+\epsilon_{ij}$$

$Y_{ij}$: Observation from the ith treatment and jth experimental unit. μ: Overall mean. $\tau_{i}$: Effect of the ith treatment. $\epsilon_{ij}$: Random error associated with the jth experimental unit receiving the ith treatment.

## 3. Results

### 3.1. Effects of AP and SP on Calf Growth Performance and Diarrhea Index

**Figure 1:**
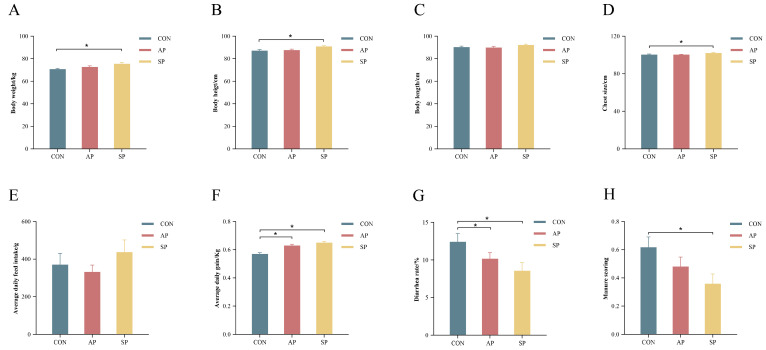
(**A**) Body weight, (**B**) body height, (**C**) body length, (**D**) chest size, (**E**) average daily intake, (**F**) average daily gain, (**G**) diarrhea rate, (**H**) manure scoring. * *p* < 0.05 compared with the CON group.

Compared to the CON, the body weight, height, and chest circumference of calves in the SP group significantly increased, rising by 6.72%, 4.27%, and 1.66%, respectively (*p* < 0.05) (Figure 1A,B,D). The average daily gain was 10.53% and 14.04% higher in the AP and SP groups compared to the CON (*p* < 0.05) (Figure 1F). Additionally, the diarrhea score in the SP group decreased by 41.94% (*p* < 0.05) (Figure 1H). Compared to the CON, the diarrhea rates in the AP and SP groups reduced by 18.12% and 30.9%, respectively (*p* < 0.05) (Figure 1G). However, no significant differences were observed in body length and average daily feed intake between the AP and SP groups and CON (*p* > 0.05) (Figure 1C,E). The detailed data for these changes are presented in Table S1.

### 3.2. Effects of AP and SP on Calf Serum Biochemistry, Antioxidant Capacity, and Immunity

**Figure 2:**
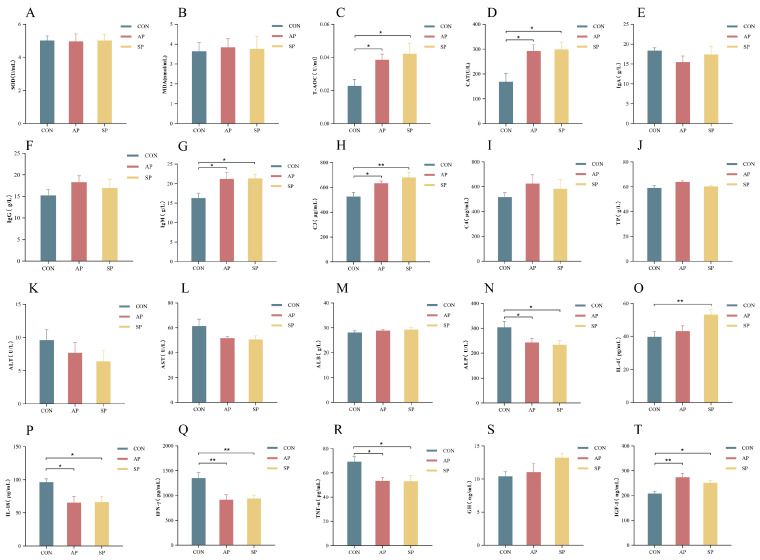
Effects of AP and SP on calf serum metabolites. (**A**) Superoxide dismutase, (**B**) Malondialdehyde, (**C**) Total antioxidant capacity, (**D**) Catalase, (**E**) Immunoglobulin A, (**F**) Immunoglobulin G, (**G**) Immunoglobulin M, (**H**) Complement component 3, (**I**) Complement component 4, (**J**) Total protein, (**K**) Alanine aminotransferase, (**L**) Aspartate aminotransferase, (**M**) Albumin, (**N**) Alkaline phosphatase, (**O**) Interleukin-4, (**P**) Interleukin-18, (**Q**) Interferon-gamma, (**R**) Tumor necrosis factor-alpha, (**S**) Growth hormone, (**T**) Insulin-like growth factor 1. * *p* < 0.05, ** *p* < 0.01; compared with the CON group.

Compared to the CON, the concentrations of T-AOC (*p* < 0.05), CAT (*p* < 0.05), IgM (*p* < 0.05), C3 (*p* < 0.01), IL-4 (*p* < 0.01), and IGF-1 (*p* < 0.05) in the SP group increased by 85.16%, 77.29%, 31.35%, 29.19%, 34.03%, and 20.69%, respectively(Figure 2C,D,G,H,O,T). In the AP group, T-AOC (*p* < 0.01), CAT (*p* < 0.001), IgM (*p* < 0.05), C3 (*p* < 0.05), and IGF-1 (*p* < 0.01) concentrations increased by 69.13%, 73.71%, 30.30%, 20.36%, and 31.71%, respectively(Figure 2C,D,G,H,T). Additionally, compared to the CON, ALP levels in the AP and SP groups decreased by 20.21% and 23.28% (*p* < 0.05), IL-18 levels decreased by 20.21% and 23.28% (*p* < 0.05), TNF-α levels decreased by 20.21% and 23.28% (*p* < 0.05), and IFN-γ levels decreased by 20.21% and 23.28% (*p* < 0.01) (Figure 2N,P,Q,R). No significant changes were observed in other indicators (*p* > 0.05). The detailed data for these changes are presented in Table S2.

### 3.3. Effects of AP and SP on Alpha and Beta Diversity of Calf Gut Microbiota

**Figure 3:**
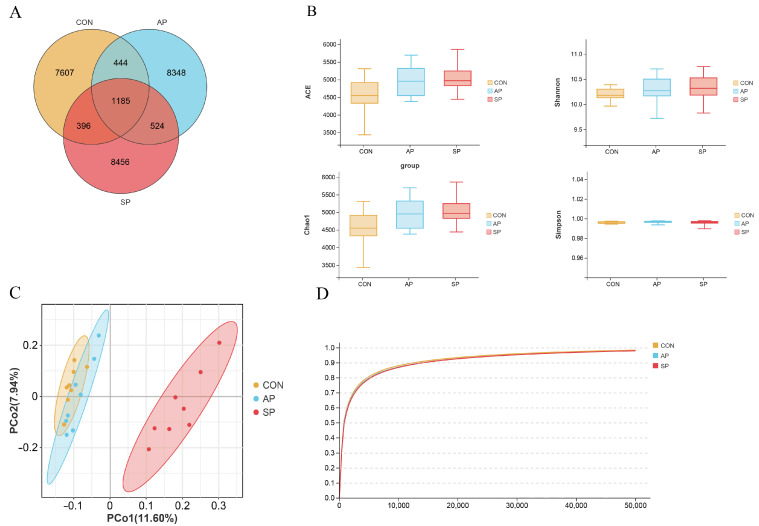
Effects of MOP on hindgut microbiota diversity. (**A**) Venn diagram of OTUs, (**B**) alpha diversity indices in each group, (**C**) PCoA plot of the gut microflora in all groups, (**D**) species observation curve in each group.

The 16S rRNA V3-V4 region of fecal samples from 24 calves was sequenced using the Illumina Novaseq 6000 platform. A total of 1185 OTUs were shared among the three treatment groups, while 7607, 8348, and 8456 OTUs were unique to the CON, AP, and SP groups, respectively (Figure 3A). In this experiment, the species accumulation curve flattened with increasing sample size, indicating that the sequencing results adequately reflected the diversity among samples within each treatment group, providing reliable data for further analysis (Figure 3D).

β-diversity, based on PCoA, measures microbiome composition similarity using unweighted Unifrac distances. The CON and SP groups were completely separated, indicating that SP had a regulatory effect on the gut microbiota. However, the CON and AP groups overlapped partially, suggesting smaller differences in data within these dimensions and potentially similar microbiota structures (Figure 3C). Overall, SP exhibited a significant regulatory effect on the gut microbiota of calves. In this study, α-diversity analysis showed that no significant differences were observed in the Shannon, Simpson, ACE, and Chao1 indices. (*p* > 0.05) (Figure 3B).

### 3.4. Effects of AP and SP on Calf Gut Microbiota

**Figure 4:**
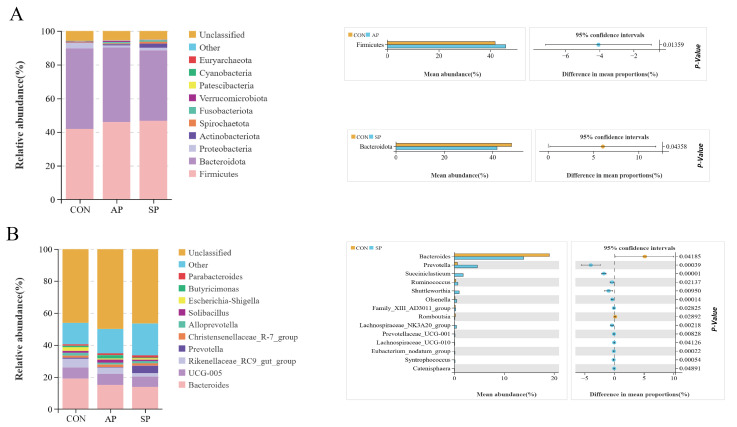
Effects of AP and SP on hindgut microbiota of calves. (**A**) Phylum level. (**B**) Genus level.

At the phylum level, the dominant phyla detected included *Firmicutes*, *Bacteroidota*, *Proteobacteria*, *Actinobacteriota*, *Spirochaetota*, *Fusobacteriota*, and *Verrucomicrobiota* (Figure 4A). Analysis showed that, compared to the CON, the abundance of *Firmicutes* increased in the AP group (*p* < 0.05), while the abundance of *Bacteroidota* decreased in the SP group (*p* < 0.05). Additionally, feeding AP and SP tended to increase the *Firmicutes* to *Bacteroidota* ratio (F/B) (Figure 4B).

At the genus level, the dominant genera detected included *Bacteroides*, *UCG-005*, *Rikenellaceae_RC9_gut_group*, *Prevotella*, *Escherichia-Shigella*, *Butyricimonas*, and *Parabacteroides* (Figure 4B). Analysis showed that, compared to the CON, the relative abundance of beneficial bacteria such as *Prevotella*, *Succiniclasticum*, *Ruminococcus*, *Shuttleworthia*, and *Oscillibacter* increased significantly in the SP group (*p* < 0.05), while the relative abundance of Bacteroides decreased (*p* < 0.05).

**Figure 5:**
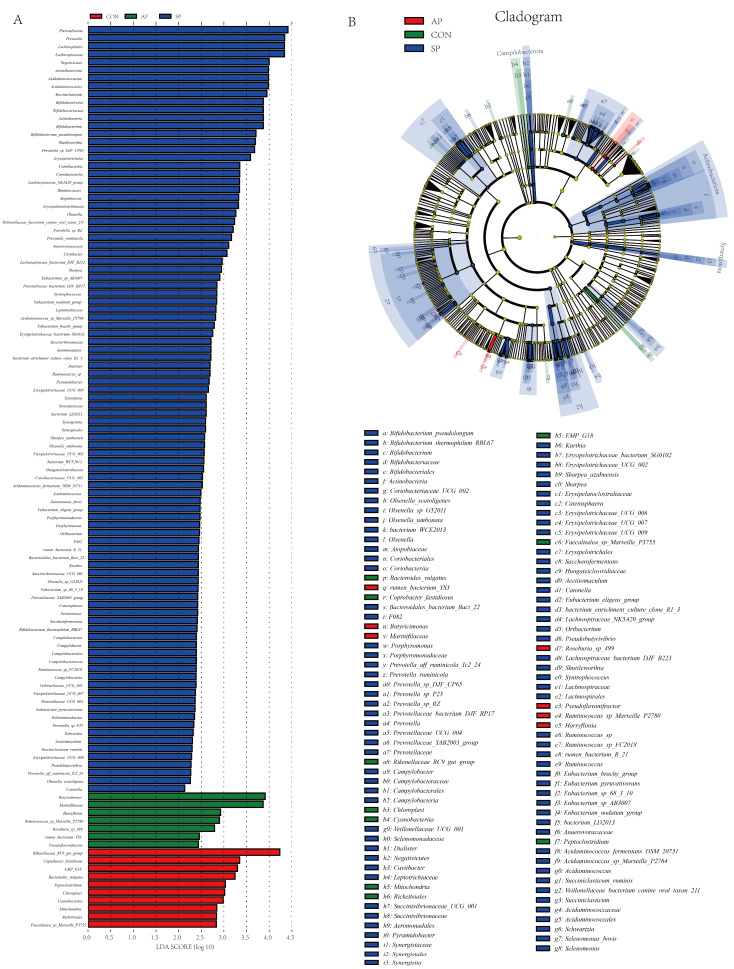
LEfSe analysis and cladogram of gut microbiota in each group.

LEfSe analysis, a tool for discovering and explaining biomarkers in high-dimensional data, was used to identify statistically different biomarkers between groups. In this experiment, with an LDA threshold of 2 and *p* < 0.05, a total of 113 differential OTUs were identified, including 3 phyla, 6 classes, 11 orders, 17 families, 44 genera, and 32 species (Figure 5). In the SP group, the abundance of beneficial bacteria such as *Prevotella*, *Lachnospiraceae*, *Negativicutes*, *Actinobacteriota*, *Acidaminococcaceae*, *Bifidobacteriaceae*, *Shuttleworthia*, *Erysipelotrichaceae*, *Coriobacteriia*, and *Ruminococcus* increased significantly (*p* < 0.05). In the AP group, the abundance of beneficial bacteria such as *Rikenellaceae_RC9_gut_group*, *Coprobacter_fastidiosus*, *EMP_G18*, *Bacteroides_vulgatus*, *Peptoclostridium*, and *Cyanobacteriia* also increased (*p* < 0.05). In the CP group, pathogenic bacteria such as *EMP_G18*, *Peptoclostridium*, and *Rickettsiales* were observed (*p* < 0.05). These results indicated that feeding calves with AP and SP could improve gut microbiota by increasing beneficial bacteria and reducing pathogenic bacteria. The phylogenetic tree in Figure 5 reveals the evolutionary relationships among microbial communities. Notably, the SP group exhibited a significant proportion of unique microbial taxa, distinguishing its microbial composition from other groups. This highlights the potential significance of specific microbial families and genera in the SP group for gut health.

### 3.5. Functional Prediction of Gut Microbiota in Calves Treated with Polysaccharides

**Figure 6:**
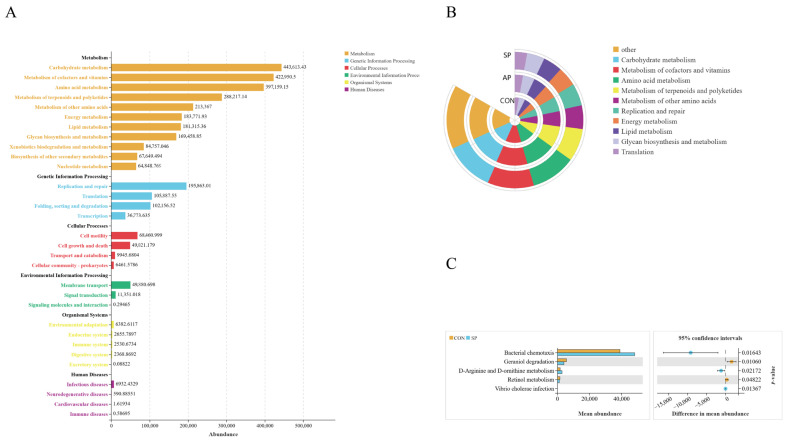
(**A**) Overview of PICRUSt2 functional distribution in the CON group. (**B**) Functional classification at KEGG Level 2. (**C**) Differential functions between two comparison groups at KEGG Level 3.

The bar plot of functional abundance in the CON classified and quantified the functions of microbial communities based on different functional categories. Functional classification was based on two levels: Level 1 and Level 2. Level 1 classification provided a broad functional overview, categorizing the functions of microbial communities into major categories. The Level l categories included metabolism, genetic information processing, cellular processes, environmental information processing, organismal systems, and human diseases. The KEGG pathway annotation results indicated that the microbiota in the CON group was primarily annotated with carbohydrate metabolism (443,613.43 ASVs), amino acid metabolism (397,159.15 ASVs), metabolism of terpenoids and polyketides (288,217.14 ASVs), metabolism of cofactors and vitamins (422,950.5 ASVs), energy metabolism (183,771.93 ASVs), and lipid metabolism (181,315.36 ASVs) (Figure 6B). The functional abundance bar plots for AP and SP were similar (Figure S2).

A circular bar plot showed the relative abundance of various metabolic functions in different sectors among the three treatment groups. The top ten biological functions included carbohydrate metabolism, metabolism of cofactors and vitamins, amino acid metabolism, metabolism of terpenoids and polyketides, metabolism of other amino acids, replication and repair, energy metabolism, lipid metabolism, glycan biosynthesis and metabolism, and translation (Figure 6B).

At KEGG Level 3, compared to the CON group, the SP group showed an increased abundance of bacterial chemotaxis (*p* < 0.05), while the CON group showed an increased pathway for geraniol degradation (*p* < 0.05). Additionally, there was an increase in the metabolism of D-arginine and D-ornithine in the SP group (*p* < 0.05) (Figure 6C).

### 3.6. Metabolomics Analysis of Calves Gut with Polysaccharides

**Figure 7:**
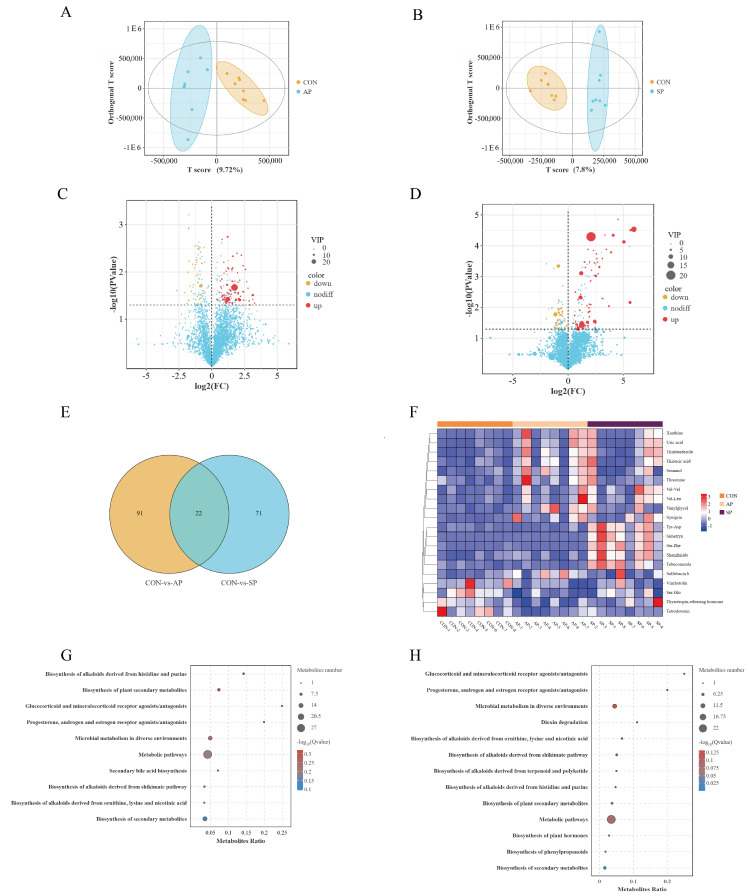
(**A**) OPLS-DA plot of CON vs. AP. (**B**) OPLS-DA plot of CON vs. SP. (**C**) Volcano plot of CON vs. AP. (**D**) Volcano plot of CON vs. SP. (**E**) Differential Venn diagram. (**F**) Heatmap of metabolite expression. (**G**) Significance bubble plot of CON vs. AP. (**H**) Significance bubble plot of CON vs. SP. Red indicates upregulation of metabolite expression; blue indicates downregulation of expression.

In the analysis of gut metabolites in calves using untargeted metabolomics, significant metabolic differences were observed between the CON and both AP and SP groups using OPLS-DA (Figure 7A,B). The modeling and predictive abilities of the OPLS-DA model were evaluated using the R2Y and Q2 parameters, representing the model’s explanatory and predictive power, respectively (Figure S1).

In the comparison between the CON and AP groups, 113 differential metabolites were identified, while 93 differential metabolites were found in the comparison between the CON and SP groups. The selection criteria for these differential metabolites were a fold change ≥ 1 and a *p*-value < 0.05. Volcano plots displayed the differential metabolites compared to the CON, with 77 metabolites upregulated in the AP group, including Sesamol, L-cysteinesulfinic acid, Vanylglycol, and uric acid, and 36 metabolites downregulated, such as beta-glycerophosphate, 5-Hydroxytryptophan, Ser-Glu, and L-cysteinesulfinic acid (Figure 7C). Compared to the CON group, 65 differential metabolites were upregulated in the SP group, including Ser-Phe, N-acetyl-d-lactosamine, Chalepensin, and Carbazole, and 28 metabolites were downregulated, such as Rubiadin, Dulcitol, Ser-Glu, and 5-Hydroxytryptophan (Figure 7D). Venn diagrams showed 91 unique differential metabolites in the comparison between the CON and AP groups and 71 unique differential metabolites in the comparison between the CON and SP groups. Additionally, 22 shared differential metabolites were identified between the two groups (Figure 7E).

To visually display the expression levels of these significant differential metabolites in each sample or group, metabolites were z-score-normalized, and heatmaps were generated using the R package. In the AP group, the expression levels of Xanthine, uric acid, thiouric acid, Sesamol, and Threonine were higher than those in the CON group. Similarly, in the SP group, the expression levels of Tyr-Asp, Ser-Phe, and Shanzhiside were relatively higher (Figure 7F).

KEGG pathway enrichment analysis was performed on the differential metabolites to explore their biological functions. The metabolites that differed between the CON and AP groups were primarily enriched in several pathways (Figure 7G). These pathways include glucocorticoid and mineralocorticoid receptor agonists/antagonists, as well as progesterone, androgen, and estrogen receptor agonists/antagonists. Additional pathways involve the biosynthesis of alkaloids derived from histidine and purine, the biosynthesis of secondary bile acids, and the biosynthesis of plant secondary metabolites. The biosynthesis of alkaloids derived from ornithine, lysine, and nicotinic acid was also enriched. The differential metabolites between the CON and SP groups were enriched in similar immune pathways, including glucocorticoid and mineralocorticoid receptor agonists/antagonists, progesterone, androgen and estrogen receptor agonists/antagonists, biosynthesis of plant secondary metabolites, biosynthesis of alkaloids derived from ornithine, lysine and nicotinic acid, and biosynthesis of alkaloids derived from histidine and purine (Figure 7H).

### 3.7. Correlation Analysis

**Figure 8:**
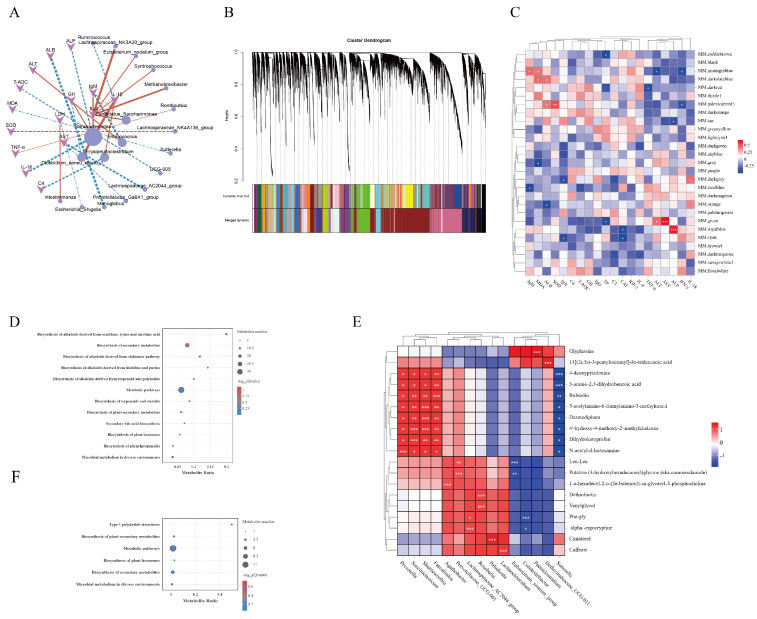
(**A**) Correlation analysis between gut microbiota at the genus level and serum indices. (**B**) Module hierarchical clustering. (**C**) Heatmap of traits and module correlations. (**D**,**F**) KEGG enrichment analysis within modules. (**E**) Correlation analysis between gut microbiota and differential metabolites. * *p* < 0.05, ** *p* < 0.01, *** *p* < 0.001.

Spearman correlation analysis was performed between genus-level microbiota and serum indices. The network analysis graph showed that microbiota that were positively correlated with IL-4 included *Candidatus_Saccharimonas* (*p* < 0.05), *Lachnospiraceae_NK3A20_group* (*p* < 0.01), and *Methanobrevibacter* (*p* < 0.01). IL-1β was positively correlated with *Clostridium sensu_stricto_1* (*p* < 0.01). *Parabacteroides* was positively correlated with IgM (*p* < 0.01) but negatively correlated with IL-18 (*p* < 0.01). At the same time, IgM was negatively correlated with *Enterococcus* (*p* < 0.001). ALB was negatively correlated with *Monoglobus* (*p* < 0.01), while AST was negatively correlated with *Lachnospiraceae_AC2044_group* (*p* < 0.01) (Figure 8A). WGCNA analysis was performed using the R package. Modules were initially divided using Dynamic Tree Cut, and similar modules were merged based on module eigengene similarity to form the final merged dynamic modules (Figure 8B). A heatmap showed that MM.greenmodule was closely related to AST (*p* < 0.001) and MM.royalbluemodule was closely related to ALP (*p* < 0.001) (Figure 8C).

Additionally, metabolites in the MM.greenmodule were enriched in KEGG pathways, including biosynthesis of alkaloids derived from ornithine, lysine, and nicotinic acid; biosynthesis of alkaloids derived from the shikimate pathway; metabolic pathways; biosynthesis of plant secondary metabolites; and microbial metabolism in diverse environments, which were the same pathways enriched in differential metabolites between the CON and AP groups (Figure 8D). Similarly, pathways enriched in MM.greenmodule, such as biosynthesis of plant secondary metabolites, metabolic pathways, biosynthesis of plant hormones, and microbial metabolism in diverse environments, were the same pathways enriched in differential metabolites between the CON and SP groups (Figure 8F). Interestingly, the D-arginine and D-ornithine metabolism pathways enriched in the SP group’s Level 3 PICRUSt2 functional predictions were related to the metabolism and synthesis of ornithine, similar to the biosynthesis pathways of differential metabolites enriched in alkaloids derived from ornithine, lysine, and nicotinic acid.

Pearson correlation analysis was performed between microbiota at the genus level and differential metabolites, and the top 20 correlations by absolute correlation coefficients were selected to create a correlation heatmap to evaluate the relationship between microbiota and metabolites (Figure 8E). *Prevotella* was positively correlated with N-acetyl-d-lactosamine (*p* < 0.001), *Succiniclasticum* was positively correlated with 4’-hydroxy-4-methoxy-2’-methylchalcone and Dihydroketoprofen (*p* < 0.001), *Shuttleworthia* was positively correlated with 5-acetylamino-6-formylamino-3-methyluracil, Desmedipham, and 4’-hydroxy-4-methoxy-2’-methylchalcone (*p* < 0.001), *Faecalitalea* was positively correlated with Rubiadin (*p* < 0.001), *Agathobacter* was positively correlated with 1-o-hexadecyl-2-o-(2e-butenoyl)-sn-glyceryl-3-phosphocholine (*p* < 0.001), *Prevotellaceae UCG-001* was positively correlated with Putative (3-hydroxyhexadecanoyl)glycine (aka commendamide) (*p* < 0.001), *Lachnospiraceae_AC2044_group* was positively correlated with α-ergocryptine (*p* < 0.001), *Roseburia* was positively correlated with Cimaterol (*p* < 0.001), and *Lachnoclostridium* was positively correlated with Caffeate (*p* < 0.001). *Eubacterium siraeum group* was negatively correlated with Leu-Leu (*p* < 0.001), *Colidextribacter* was negatively correlated with Phe-gly (*p* < 0.001), *Defluviitaleaceae UCG-011* was negatively correlated with 13[(2r,3s)-3-pentyloxiranyl]-8z-tridecenoic acid (*p* < 0.001), and *Sutterella* was negatively correlated with 4-deoxypyridoxine and 3-amino-2,3-dihydrobenzoic acid (*p* < 0.001).

## 4. Discussion

As safe and effective natural bioactive substances, AP and SP have the potential to replace antibiotics in calf farming to alleviate neonatal calf enteritis [14,15]. Therefore, their effects on the growth performance, serum metabolites, gut microbiota, and metabolomics of newborn calves were studied.

Diarrhea caused by enteritis is one of the main causes of death in neonatal calves within the first few days after birth, leading to significant economic losses in calf farming [22]. This study is the first to investigate the effects of these substances on the intestinal health and growth development of neonatal Holstein calves. The results showed that the addition of SP increased calf body weight and height and reduced the diarrhea rate and diarrhea score. This may have been related to the regulation of enzyme activity by these substances [23]. The addition of polysaccharides is highly effective in reducing diarrhea rates in piglets [24], but its application in ruminants has been rarely reported. Previous studies have shown that polysaccharides can promote bacterial growth by regulating gut flora and immunity, thereby improving animal performance [25]. They are reported to be effective in treating diarrhea and improving animal diseases caused by bacterial infections, while also enhancing body weight and immunity [26], which is consistent with our results.

Changes in calf serum composition could reflect animal metabolic changes [27]. T-AOC levels indicate the body’s health status; higher T-AOC levels usually signify strong antioxidant capacity, helping to resist damage caused by oxidative stress. CAT is an important antioxidant enzyme that decomposes hydrogen peroxide into water and oxygen, reducing cell oxidative damage. Zhang et al. showed that adding glycyrrhizin polysaccharides to the diet of broilers significantly increased serum T-AOC concentrations [28]. Studies have shown that lentinan has potential effects by inhibiting oxidative stress, manifesting as increased CAT activity [29]. In this study, AP and SP were found to increase CAT and T-AOC levels in the serum of neonatal calves, consistent with the above findings. The reason for this may be that, after feeding AP and SP to neonatal calves, polysaccharides directly participated in free radical reactions, acting as free radical scavengers and terminating the chain reaction of free radicals.

As a pentameric antibody, IgM is secreted by B cells and has the capability to activate the complement system, exert lysosomal activity, and neutralize viruses [30]. C3 is a central component of the complement system, involved in the activation of the classical pathway, the alternative pathway, and the lectin pathway. The activation products of C3 play key roles in regulating immune responses, promoting phagocytosis, and clearing pathogens [31]. Liu et al. found that feeding Chinese herbal polysaccharides to laying hens significantly increased serum IgM levels, thereby enhancing immunity [32]. Ficus polysaccharides and polygonum polysaccharides have been shown to affect the immune parameters of crucian carp, significantly increasing the content of C3 [33]. The results of this study indicated that AP and SP significantly increased the levels of IgG and C3 in calf serum, suggesting that these polysaccharides could stimulate the immune function of calves. IgG can activate the complement system through the classical pathway, ultimately leading to the activation and cleavage of C3 [34]. This enhancement in immunity could reduce the incidence of diseases in neonatal calves.

Widely distributed in the liver, bone, gut, kidney, and placenta of animals, ALP is an enzyme primarily used for the diagnosis and differential diagnosis of skeletal and hepatobiliary system diseases. Jian et al. found that feeding purple sweet potato polysaccharides to mice significantly reduced serum ALP levels [35]. The results of this study indicated that AP and SP significantly reduced the ALP content in calf serum, suggesting that these polysaccharides might have had positive effects on calf liver function and bone health. TNF-α, IFN-γ, and IL-18 are three important pro-inflammatory cytokines that play key roles in immune responses and inflammation. Polysaccharides from *Scutellaria barbata* D. Don were shown to reduce the levels of inflammatory cytokines such as TNF-α, IFN-γ, and IL-18 in the colon [36]. This study is consistent with the aforementioned findings, indicating that these three cytokines play important roles in immune regulation, inflammation response, and anti-infection.

Cell proliferation and differentiation are promoted by IGF-1 binding to the IGF-1 receptor, which activates the PI3K/AKT and MAPK signaling pathways [37]. Studies have shown that in early-weaned piglets, adding different concentrations of mulberry leaf polysaccharides to the diet significantly increased IGF-1 levels compared to the control group. The results of this experiment are consistent with the above findings.

The gut is a crucial environment for microbial growth in organisms, essential for maintaining a stable microbiota and animal health [38]. Many studies have shown that polysaccharides could regulate gut bacteria [39,40]. Firmicutes and Bacteroidetes were the most representative gut bacteria at the phylum level in calves. A decrease in Firmicutes indicated gut dysfunction [41]. Another study found that when enteritis occurred, the gut microbiota became imbalanced, and supplementing with polysaccharides alleviated enteritis [42]. During this process, the F/B ratio tended to increase [43]. In this experiment, the F/B ratio in newborn calves fed AP and SP showed an upward trend, similar to the aforementioned results, indicating that AP and SP had positive effects on stabilizing the gut microbiota and anti-diarrheal effects. Polysaccharides have been proven to play significant roles in gut health, and most of these polysaccharides cannot be digested and absorbed by the gut. However, they can be digested and processed by the gut microbiota, stimulating the growth of microbial communities and the production of metabolites, which have positive effects on the host, especially in treating gut inflammation [44].

This experiment showed that SP affected the relative abundance of bacterial genera. *Prevotella* is a Gram-negative bacterium that may influence health by regulating the immune system. They can activate specific immune cells, such as Th17 cells, which play a vital role in maintaining gut barrier function and preventing pathogen invasion [45]. Additionally, *Prevotella* has been reported to promote the degradation and absorption of proteins and fibers in ruminants [46]. *Succiniclasticum* is a bacterial genus that primarily converts succinate to propionate, an important short-chain fatty acid in the gut. Propionate is beneficial to the host as an energy source and is associated with various health benefits, including improved glucose metabolism and lower cholesterol levels [47]. Higher levels of *Ruminococcus* help maintain a healthy gut barrier. Butyrate is one of the SCFAs produced by *Ruminococcus*, known to support the integrity of the gut epithelium and reduce inflammation [48]. *Subdoligranulum* is a known butyrate-producing bacterium [49]. Microbial-derived butyrate has been reported to promote host intestinal epithelial barrier function [50].

In the LEfSe analysis, *Peptoclostridium* and *Rickettsiales* had higher abundances in the CP group, while they were absent in the AP and SP groups. *Peptoclostridium difficile* is a related species that can produce toxins A and B, leading to severe enteritis and diarrhea [51]. Infection with *Rickettsiales* in cattle usually results in health problems, including reduced milk production and slower growth rates, leading to decreased productivity. In this study, the increase in *Prevotella*, *Succiniclasticum*, *Ruminococcus*, and *Shuttleworthia*, along with the decrease in *Peptoclostridium* and *Rickettsiales*, suggested that SP regulated gut microbial balance, increasing the abundance of beneficial bacteria while reducing the abundance of pathogenic bacteria. However, the specific mechanisms by which AP and SP affect the composition of gut bacteria in calves still require further investigation.

The effects of AP and SP on the metabolic characteristics of gut content were analyzed using LC/MS. Metabolites were screened, and the main differential metabolites identified included phenols and their derivatives, amino acids and their derivatives, sugars and their derivatives, and plant metabolites. Sesamol, 5-hydroxytryptamine, Dulcitol, and Chalepensin were important metabolites in this study.

Phenolic compounds regulate immune response by inhibiting the synthesis and gene expression of pro-inflammatory cytokines [52]. Sesamol has powerful antioxidant properties, capable of neutralizing free radicals and reducing oxidative stress. It also exhibits anti-inflammatory effects by inhibiting the synthesis of pro-inflammatory cytokines, such as IL-1β and TNF-α, and modulating the NF-κB and MAPK signaling pathways [53]. Amino acids and their derivatives, such as 5-hydroxytryptamine, play various roles in regulating immune cell functions. 5-hydroxytryptamine regulates cytokine secretion by monocytes and macrophages through its receptors. Studies have shown that 5-hydroxytryptamine inhibits the release of TNF-α and IL-1β by activating serotonin receptors [54]. Sugars and their derivatives directly exert antitumor effects by inducing cell cycle arrest and apoptosis. Most polysaccharides modulate the host immune system and indirectly inhibit tumors by activating non-specific or specific immune responses [55]. Adding Dulcitol has been shown to alleviate LPS-induced intestinal barrier damage in piglets by maintaining intestinal integrity, inhibiting the TLR4/NF-κB signaling pathway and apoptosis, and regulating gut microbiota [32]. Chalepensin exhibits inhibitory effects on various bacteria and fungi, particularly pathogenic organisms like *Staphylococcus aureus* and *Candida albicans* [56].

Compared to the CON group, the differential metabolites in the AP and SP groups were enriched in pathways including glucocorticoid and mineralocorticoid receptor agonists/antagonists, progesterone, androgen and estrogen receptor agonists/antagonists, biosynthesis of alkaloids derived from ornithine, lysine, and nicotinic acid, and biosynthesis of plant secondary metabolites. Studies have shown that glucocorticoid and mineralocorticoid receptors exert their effects through rapid non-genomic mechanisms, including physical and chemical interactions with cell membranes and actions mediated by membrane-bound and cytosolic GRs [57]. These mechanisms influence anti-inflammatory, anti-allergic, and anti-shock functions. Low doses of androgen receptor antagonists or estrogen receptor agonists significantly alter skeletal muscle function. Although the biosynthesis of alkaloids derived from ornithine, lysine, and nicotinic acid has not been fully elucidated, alkaloids are known to play important roles in anti-inflammatory, antiviral, antibacterial, and anticancer activities [58]. Plant secondary metabolites exert immunosuppressive effects by regulating complex immune systems and multiple molecular targets. These metabolites, including phenols, flavonoids, chalcones, flavanones, terpenes, alkaloids, and glycosides, exhibit significant immunosuppressive and anti-inflammatory activities in experimental models [59]. Additionally, differential metabolites in the AP group are enriched in the secondary bile acid biosynthesis pathway; deoxycholic acid and lithocholic acid have been shown to inhibit the proliferation of Clostridium difficile and regulate metabolic and immune responses [60].

Intestinal microbiota and their metabolites can influence immune function [28,61]. This study showed that IL-1β was significantly positively correlated with Clostridium_sensu_stricto_1, consistent with Yi et al.’s findings [62]. IL-1β promotes the expression of inflammatory genes by binding to IL-1 receptors and activating downstream signaling pathways, such as NF-κB and MAPK pathways. Additionally, supplementation with Parabacteroides distasonis alleviates metabolic dysfunction and reduces inflammation markers [63]. This study showed a positive correlation between Parabacteroides and IgM, suggesting a regulatory role of Parabacteroides on IgM.

The WGCNA analysis further revealed that AST and ALP were significantly positively correlated with modules MM.greenand and MM.royalblue, respectively. Many of the metabolic pathways enriched in these modules overlap with those of the differential metabolites, mostly related to the biosynthesis of alkaloids derived from amino acids and other metabolites [64]. Alkaloids have been shown to inhibit bacterial growth through various mechanisms, including inhibiting bacterial nucleic acid and protein synthesis, altering bacterial cell membrane permeability, disrupting cell membranes and cell walls, and inhibiting bacterial metabolism. ALP has been demonstrated to significantly alleviate inflammatory conditions in various experimental models [35], including colitis, liver failure, and ischemia–reperfusion injury in the kidneys and heart. ALP exerts its effects by regulating TLR4-related signaling and cytokine overexpression, improving barrier tissue dysfunction and oxidative stress [35].

The correlation analysis of gut differential metabolites and microbiota revealed that *Prevotella’*s positive correlation with N-acetyl-d-lactosamine suggests their involvement in common metabolic pathways, especially in carbohydrate metabolism. *Prevotella*, a genus of Gram-negative bacteria, is involved in carbohydrate metabolism and has been linked to various beneficial health outcomes [65]. Studies show that dietary patterns, particularly those rich in fiber and plant-based foods, influence the abundance of *Prevotella* in the gut microbiota [66]. *Prevotella* species can break down complex carbohydrates, such as N-acetyl-d-lactosamine. N-acetyl-d-lactosamine is an important carbohydrate, potentially utilized and transformed through *Prevotella’*s metabolic activities [66]. Similarly, the *Lachnospiraceae* family plays a crucial role in fermenting dietary fibers into short-chain fatty acids, which benefits gut health [67]. The positive correlation of *Lachnospiraceae_AC2044_group* with α-ergocryptine suggests its involvement in alkaloid metabolism. α-ergocryptine, an alkaloid, can be effectively metabolized by this bacterial group, indicating their potential role in the biotransformation of alkaloids [68].

## 5. Conclusions

The findings indicate that both AP and SP effectively mitigated calves’ diarrhea. Compared to the CON, the diarrhea rates in the AP and SP groups were reduced by 18.12% and 30.9%, respectively. They achieved this by modulating the gut microbiota, enhancing the relative abundance of beneficial bacteria while diminishing that of pathogenic bacteria. Moreover, AP and SP influenced pathways associated with immune response and inflammation, thereby alleviating intestinal inflammation. SP exhibited superior efficacy compared to AP, although further investigation is warranted to elucidate the precise mechanisms involved. Overall, AP and SP are proposed as innovative additives for the prevention of diarrhea in calves.

## Figures and Tables

**Table 1:** Composition and nutrient level of starters and milk replacer (dry matter basis).

Items	Milk Replacer	Starters
Ingredients		
Corn		49
Soybean meal		20
Wheat middlings		7.5
Bran		7.0
Expanded soybean		3.5
Corn gluten meal		3.5
Corn germ meal		5
Premix ^1^		4.5
Total		100
Nutrition level ^2^		
Dry matter	95.61	87.31
Crude protein	21.21	23.82
Ether extract	16.66	3.43
Neutral detergent fiber	4.02	25.57
Acid detergent fiber	2.11	11.74
Ash	5.51	6.13
Calcium	0.47	0.91
Phosphorus	0.27	0.51

Note: ^1^ This premix provided the following per kg: Cu 200~500 mg, Fe 1500~2500 mg, Mn 1000~2000 mg, Zn 1000~2500 mg, VA 200,000~370,000 IU, VD 3,250,000~1,250,000 IU, VE > 750 mg, P 5~50 mg, Se 5~15 mg, Co 5~15 mg, Ca 100~160 g. ^2^ Nutrient levels were measured values.

## Data Availability

The original contributions presented in the study are included in the article/supplementary material, further inquiries can be directed to the corresponding author.

## Supplementary Materials

The following supporting information can be downloaded at https://www.mdpi.com/article/10.3390/ani15040485/s1, Figure S1: (A) Permutation test plot of OPLS-DA for CON vs. AP. (B) Permutation test plot of OPLS-DA for CON vs. SP. Figure S2: (A) Overview of PICRUSt2 functional distribution in the AP group. (B) Overview of PICRUSt2 functional distribution in the SP group. Table S1. The effects of AP and SP on the growth performance of Holstein calves. Table S2. Effects of AP and SP on Serum Biochemistry, Antioxidant Capacity, and Immunity in Holstein Calves.
