# Supplementary material for: Microbiomic and Metabolomic Insights into the Mechanisms of Alfalfa Polysaccharides and Seaweed Polysaccharides in Alleviating Diarrhea in Pre-Weaning Holstein Calves
Source: Animals (Basel). 2025 Feb 8;15(4):485. doi: 10.3390/ani15040485 (PMC11851682; doi:10.3390/ani15040485)
Supplement: Supplementary file 1 [file animals-15-00485-s001.zip › animals-3419718-supplementary.pdf]

Table S1. The effects of AP and SP on the growth performance of Holstein calves.

Items	Groups			SEM	*P*-value
	CON	AP	SP		
Body weight/kg	70.67^{c}	72.71^{ac}	75.42^{a}	0.72	0.021
Body heigt/cm	87.17^{b}	87.7^{b}	90.89^{a}	0.59	0.014
Body length/cm	90.29	89.9	92.22	0.52	0.138
Chest size/cm	100.33^{b}	100.33^{b}	102^{a}	0.33	0.045
Average daily gain/kg	0.57^{b}	0.63^{b}	0.65^{a}	0.01	0.001
Manure scoring	0.62^{a}	0.48^{ab}	0.36^{b}	0.04	0.048
Diarrhea rate/%	12.42^{a}	10.17^{b}	8.57^{b}	＜0.01	＜0.01
Average daily feed intake/g	370.66	332.28	437.44	32.02	0.354

Note：Peer data superscripts with different lowercase letters indicate significant differences (P < 0.05), while the same or no letters indicate no significant differences (P > 0.05).

Table S2. Effects of AP and SP on Serum Biochemistry, Antioxidant Capacity, and Immunity in Holstein Calves.

Items	Groups			SEM	*P*-value
	CON	AP	SP		
SOD(U/mL)	5.02	4.97	5.02	0.2	0.994
MDA（nmol/mL）	3.64	3.84	3.76	0.27	0.957
T-AOC（U/L）	22.77^{b}	38.51^{a}	42.16^{a}	2.83	0.023
CAT(U/L)	168.43^{b}	292.58^{a}	298.61^{a}	22.25	0.019
IgA（g/L）	18.39	15.47	17.4	0.83	0.367
IgG（g/L）	15.26	18.33	16.96	0.95	0.447
IgM（g/L）	16.27^{b}	21.2^{a}	21.37^{a}	0.93	0.022
C3（μg/mL）	526.39^{b}	633.55^{a}	680.06^{a}	24.44	0.014
C4（μg/mL）	516.01	624.85	583.76	34.86	0.444
TP（g/L）	59.11^{a}	63.99^{a}	60.38^{ab}	0.94	0.068
ALT（U/L）	9.60	7.69	6.40	0.92	0.386

AST（U/L）	61.41	51.63	50.69	2.28	0.104
ALB（g/L）	28.11	28.88	29.26	0.38	0.506
ALP（U/L）	304.42^{a}	242.89^{b}	233.55^{b}	12.35	0.036
IL-4（pg/mL）	39.73^{b}	43.24^{b}	53.25^{a}	2.16	0.019
IL-18（pg/mL）	96.48^{a}	65.46^{b}	66.24^{b}	5.52	0.025
IFN-γ（pg/mL）	1352.91^{a}	917.91^{b}	939.2^{b}	70.01	0.007
TNF-α（pg/mL）	69.16^{a}	53.33^{b}	53.09^{b}	2.86	0.023
GH（ng/mL）	10.42^{b}	11.06^{ab}	13.22^{a}	0.54	0.059
IGF-1（ng/mL）	207.99^{b}	273.94^{a}	251.02^{a}	8.98	0.005

Note：Peer data superscripts with different lowercase letters indicate significant differences ($P < 0.05$), while the same or no letters indicate no significant differences ($P > 0.05$).

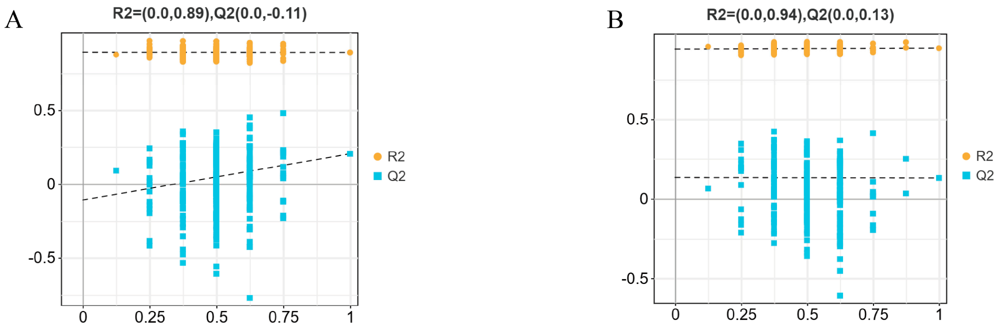

Figure S1. (A) Permutation test plot of OPLS-DA for CON vs AP. (B) Permutation test plot of OPLS-DA for CON vs SP.

A

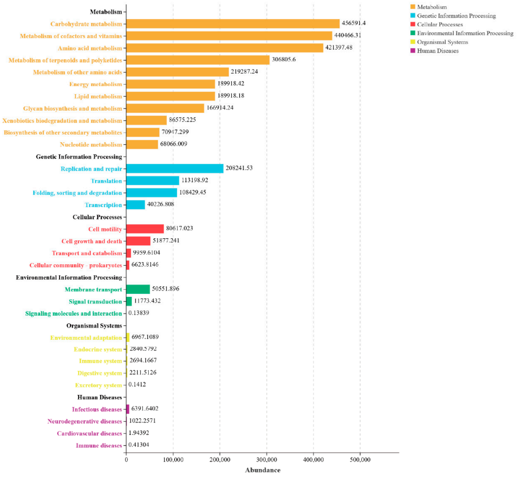

B

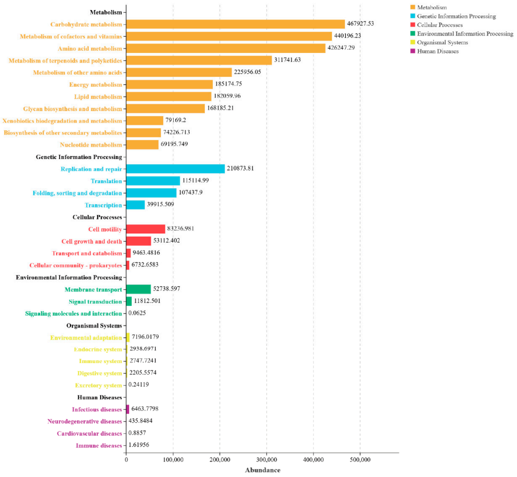

Figure S2. (A) Overview of PICRUSt2 functional distribution in the AP group. (B) Overview of PICRUSt2 functional distribution in the SP group.
